# Graph Embedding Based Novel Gene Discovery Associated With Diabetes Mellitus

**DOI:** 10.3389/fgene.2021.779186

**Published:** 2021-11-25

**Authors:** Jianzong Du, Dongdong Lin, Ruan Yuan, Xiaopei Chen, Xiaoli Liu, Jing Yan

**Affiliations:** ^1^ Zhejiang Hospital, Hangzhou, China; ^2^ Zhejiang Provincial Key Lab of Geriatrics, Zhejiang Hospital, Hangzhou, China

**Keywords:** diabetes mellitus, graph embedding, novel gene discovery, molecular network, disease gene prediction

## Abstract

Diabetes mellitus is a group of complex metabolic disorders which has affected hundreds of millions of patients world-widely. The underlying pathogenesis of various types of diabetes is still unclear, which hinders the way of developing more efficient therapies. Although many genes have been found associated with diabetes mellitus, more novel genes are still needed to be discovered towards a complete picture of the underlying mechanism. With the development of complex molecular networks, network-based disease-gene prediction methods have been widely proposed. However, most existing methods are based on the hypothesis of guilt-by-association and often handcraft node features based on local topological structures. Advances in graph embedding techniques have enabled automatically global feature extraction from molecular networks. Inspired by the successful applications of cutting-edge graph embedding methods on complex diseases, we proposed a computational framework to investigate novel genes associated with diabetes mellitus. There are three main steps in the framework: network feature extraction based on graph embedding methods; feature denoising and regeneration using stacked autoencoder; and disease-gene prediction based on machine learning classifiers. We compared the performance by using different graph embedding methods and machine learning classifiers and designed the best workflow for predicting genes associated with diabetes mellitus. Functional enrichment analysis based on Human Phenotype Ontology (HPO), KEGG, and GO biological process and publication search further evaluated the predicted novel genes.

## Introduction

Diabetes mellitus is a chronic disease where the blood sugar in patients is abnormally elevated because of the underproductive pancreas or the ineffective response toward insulin (Kharroubi and Darwish, 2015). According to the global diabetes map (ninth edition) published by the International Diabetes Federation (IDF) in 2019 (Cho et al., 2018), the number of diabetic patients worldwide is increasing, with an average global growth rate of 51%. There are currently 463 million diabetic patients. According to the growing trend, there will be 700 million diabetic patients worldwide by 2045 (Cho et al., 2018). Diabetes mellitus and its multiple complications have largely increased the risk of mortality, blindness, and kidney failure of patients, and posed a heavy burden on human society. It is urgent to investigate the disease mechanisms and find more effective cures.

There are different types of diabetes: type 1 diabetes (T1D), type 2 diabetes (T2D), gestational diabetes and other types (Geerlings and Hoepelman, 1999; Kharroubi and Darwish, 2015). For different types of diabetes, the causes and risk factors vary. Type 1diabetes is an autoimmune disease, where the insulin-producing cells in the pancreas are attacked by the immune system of patients. The pathogenesis of type 1 diabetes is still unclear, but some researchers think it is caused by a combination of genetic and environmental factors. The genome-wide association studies (GWAS) have identified over 60 susceptibility loci for T1D (Systematic evaluation of genes and genetic variants associated with Type 1 diabetes susceptibility). And post-GWAS functional analyses (Shabalin, 2012; Westra et al., 2013; Fagny et al., 2017; Wang et al., 2019a; van der Wijst et al., 2020) such as expression quantitative trait loci (eQTL) analysis have been performed to infer the underlying causal genes (Nyaga et al., 2018). Cells become resistant to insulin in type 2 diabetes, resulting in higher demand for insulin. However, the dysfunction of pancreatic β cells decreases secretion of insulin, leading to evaluated blood sugar levels in patients. The pathogenesis of T2D is also unclear, but the genetic studies of T2D provided novel susceptibility loci and candidate genes. Similarly, the mechanisms of other types of diabetes are also not clear. It is urgent to discover genes associated with diabetes mellitus to find therapeutic targets and improve diagnoses (Kharroubi and Darwish, 2015).

There have been intense efforts to predict genes associated with complex diseases in recent years (Ghiassian et al., 2015; Peng et al., 2017; Agrawal et al., 2018; Cheng et al., 2019; Wang et al., 2020). GWASs can directly reveal the associations between genome variants and diseases (Zhu et al., 2016a; Zhu et al., 2016b; Visscher et al., 2017; Gallagher and Chen-Plotkin, 2018; Visscher and Goddard, 2019). However, most GWAS SNPs locate in non-coding regions, i.e., intronic or inter-genetic regions, leading to a limited discovery of disease genes. Functional analysis, such as eQTL analysis (Wang et al., 2021a; Wang et al., 2021b), can further translate GWAS signals to functional genes through measuring the regulation pattern between genomic variations (genotypes) and transcriptome variations (gene expression level). These statistical methods have achieved tremendous success in discovering disease-associated genes. And these discoveries have also been recorded in biological databases such as DisGeNet (Piñero et al., 2015; Piñero et al., 2016; Piñero et al., 2020). However, these methods mostly are based on simple “gene-disease” associations and ignore the underlying functional collaborations among genes.

With the development of molecular networks, such as protein-protein interaction (PPI) networks and gene regulatory networks, it is feasible to investigate disease genes based on gene networks (Peng et al., 2021a). Under the hypothesis of guilt–by–association (GBA), the novel disease-associated genes can be predicted by measuring the neighborhood structures of known disease genes. In recent years, there have been many network-based methods emerging as powerful tools for disease-gene prediction (Wang et al., 2019b; Wang et al., 2019c; Yang et al., 2019). The task of disease-gene prediction can be considered as a classification problem in machine learning. There are two types of classification in disease-gene prediction based on the types of entity the methods aim to predict. One is node classification, where genes in the gene network can be separated into two groups: known disease-genes and unlabeled genes, and the prediction methods aim to give a rank to unlabeled genes based on the prediction model. Top-ranked genes will be predicted as novel disease genes. Methods such as PRINCE (Vanunu et al., 2010), VAVIEN (Erten et al., 2011), and N2A-SVM (Peng et al., 2019a) belong to this category. The other type of classification in disease-gene prediction is edge classification, also called link prediction. In this category, genes and diseases both exist in the network as nodes, which comprise a heterogeneous graph. The prediction methods learn features from known disease-gene edges and predict novel disease-gene links. The feature of a disease-gene link is combined from a pair of node features. Methods such as RWRH (Li and Patra, 2010) and RWPCN (Yang et al., 2011) belong to this category.

From the aspect of features extracted from the network, the disease-gene prediction methods can be separated into handcrafted feature-based methods and automatic feature representation-based methods. In the first category, methods engineered features for nodes in biological networks, such as using node degree, graphlet degree, common neighbors, shortest path length meta-paths, etc. However, methods relying on direct neighborhood counting can only capture the local network structure while ignoring the global structure. To overcome this issue, Xu et al. proposed a method by integrating multiple topological features to predict disease genes (Xu and Li, 2006). In their methods, they expanded the neighbors of a seed by considering 2-hop neighbors. Besides the network topological structure, some methods integrated more biological data as features. DERanking (Nitsch et al., 2010) incorporated differential expression in features. BRIDGE (Chen et al., 2013) integrated multiple data sources besides the PPI network, such as gene expression, gene ontology (GO), and the KEGG database. DiGI (Tran et al., 2020) used gene co-expression network, functional pathways, PPI network, and other cofunction networks in feature engineering. Although these methods based on handcrafted features have achieved tremendous success in multiple fields, there needs a lot of domain knowledge and it may also introduce biases with manually engineered features.

In recent years, graph embedding learning methods emerged as powerful tools for extracting the latent features from networks. Graph embedding is also known as graph representation learning, aiming at mapping large and sparse graph data into low-dimensional dense feature vectors. There are matrix factorization-based graph embedding methods [such as IMC (Natarajan and Dhillon, 2014) and PCFM (Zeng et al., 2017)], and also methods based on skip-gram based neuron networks [such as LINE (Tang et al., 2015), DeepWalk (Perozzi et al., 2014), and Node2Vec (Grover and Leskovec, 2016)], and graph neuron networks [such as graph convolutional network (Wu et al., 2020)]. These techniques have been widely used in bioinformatics applications such as the discovery of antibiotics (Stokes et al., 2020), disease genes (Peng et al., 2021b), disease modules (Wang et al., 2020), drug targets (Peng et al., 2021c), drug side-effects (Han et al., 2021), RNA-targets (Peng et al., 2019b), molecular network edges (Perozzi et al., 2014; Ribeiro et al., 2017; Peng et al., 2021d), etc. However, there has been a lack of research on discovering genes associated with diabetes mellitus using cutting-edge graph-embedding techniques. In this study, we designed a computational framework based on graph embedding approaches to discover novel genes associated with diabetes mellitus without distinction between diabetes types. We first extracted gene features from a PPI network. During this phase, we compared three cutting-edge graph embedding methods, i.e., LINE (Tang et al., 2015), DeepWalk (Perozzi et al., 2014), and Node2Vec (Grover and Leskovec, 2016). Next, we applied a stacked auto-encoder to further process the node embeddings into lower-dimensional space. Finally, we used widely-used machine learning classifiers for the task of gene prediction. In the experiments, we evaluated the performance of our model by using five-fold cross-validation, and we also compared the performance using various graph embedding methods, hyper-parameters, and machine learning classifiers.

## Methodology

**FIGURE 1:**
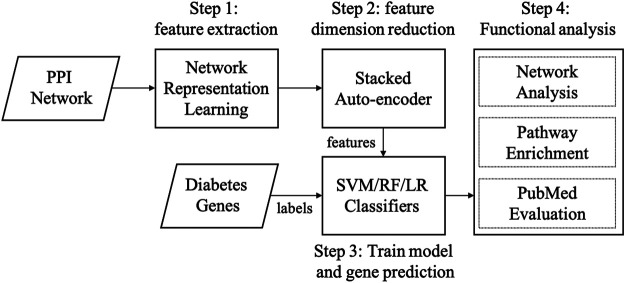
Workflow of our method. Abbreviations: SVM: supporting vector machine, RF: random forest, LR: logistic regression.

There are three main steps in our graph embedding based diabetes-gene prediction model: 1) we used three cutting graph embedding methods, i.e., LINE, DeepWalk, and Node2Vec, to extract node features from a PPI network; 2) A three-layer stacked autoencoder was applied to further reduce feature dimension and automatic feature extraction; 3) disease gene prediction using support vector machine (SVM) (Chang and Lin, 2011), and other two widely-used classifiers (random forest and logistic regression) were compared. Four metrics (AUPRC, AUROC, ACC, and F1 score) were used to measure the performance in five-fold cross-validation. Functional enrichment and network analysis were applied for evaluation. The workflow of our method is shown in Figure 1.

### Extract Features From PPI Network Based on Graph Embedding

To extract the latent feature from PPI network, we adopt three cutting-edge graph embedding methods: Node2vec, DeepWalk, and LINE, and compared their performance in the task of predicting genes associated with diabetes mellitus. DeepWalk draws on the idea of the word2vec algorithm. Word2vec is a commonly used word embedding method in natural language learning (NLP). It describes the co-occurrence relationship between words and words through the sentence sequence in the corpus and then learns the vector representation of words based on skip-gram neuronal network model. The DeepWalk algorithm is similar to word2vec and uses the co-occurrence relationship between nodes in the graph to learn the vector representation of nodes. DeepWalk uses random walk to sample paths with fixed lengths. The paths are consisted of randomly visited nodes and are similar to sentences in NLP. And then word2vec is used to learn the co-occurrence relationship of nodes based on skip-gram neuronal network model. The weights on the hidden layer of skip-gram model will be the latent features.

Node2vec is a graph embedding method improved based on DeepWalk. The novel part of Node2vec is that it uses a biased random walk process to generate random paths. The hyperparameters *p* and *q* are used to control the directions of random walk in consonance with breadth-first search (BFS) or depth-first search (DFS) in the PPI network. Parameter *p* determines the process of revisiting the nodes within random walk and *q* affects the possibility of capturing local or global nodes. Compared to DeepWalk, Node2vec provides more various elements, and particularly, if the value of *p* and *q* both equal 1, these two algorithms are the same.

LINE is also a method based on the assumption of neighborhood similarity, except that LINE uses BFS to construct neighborhoods while DeepWalk uses DFS to construct neighborhoods. LINE also takes into account the first-order and second-order similarities between nodes and can be applied to various types of networks and large-scale networks. However, some vertices have few adjacent points, which leads to insufficient learning of embedding vectors and insufficient use of high-level information.

### Feature Regeneration and Reduction Using Stacked Autoencoder

Autoencoder is an unsupervised artificial neural network that can automatically extract latent features from data. Autoencoder has been successfully applied in many applications, such as speech recognition, self-driving cars, human gesture detection, etc. The autoencoder structure is composed of three parts: the input layer, the hidden layer, and the output layer, which correspond to the encoder, bottleneck and decoder respectively. Among them, the encoder is responsible for selecting key features from the data, and the decoder is responsible for recreating the original data using key components. Since the number of hidden layer nodes is less than the number of input nodes, the autoencoder can reduce the data dimension by retaining only the features needed to reconstruct the data. The autoencoder is also a feed-forward network, which can be trained using the same procedure as the feed-forward network. Although Autoencoder has the same input and output, it also has a certain degree of loss, so autoencoder is also called lossy compression technology.

Since there are complicated relationships within the elements in some data sets, only one autoencoder cannot meet the requirements. To reduce the dimensionality of the input features, a single autoencoder may not be able to complete it. In response to this situation, the stacked autoencoder was proposed. As the name suggests, stacked autoencoders are multiple autoencoders stacked on top of each other. The specific process of the stacked autoencoder method is described as follows: First, given the initial input, train the first-layer autoencoder in an unsupervised way to reduce the reconstruction error to the set value. Second, take the output of the hidden layer of the first autoencoder as the input of the second autoencoder, and use the same method as above to train the autoencoder. Third, repeat the second step until all autoencoders are initialized. Finally, use the weights of the hidden layer of the last stacked autoencoder as the final features.

### Machine Learning Classifiers Used for Disease Gene Prediction

After the process of network representation learning and feature denoising, we apply classification methods for the final prediction task. Three widely-used machine learning algorithms were used for predicting genes associated with diabetes mellitus: support vector machine (SVM), Logistic regression, and Random Forest. Logistic regression models the relationship between predictor variables and a categorical response variable. Given feature vector ***x*** and the label y∈{0,1} of each sample, the logistic regression models feature ***x*** and the probability of *y* by Eq. 1, where ***w*** represents weights and b represents bias. This equation means the log odds of prediction *y =* 1 equals linear regression of input feature ***x***. The parameters ***w*** and *b* can be estimated by maximum likelihood estimation.

$$w^{T}x+b=ln\frac{p\left(y=1|x\right)}{1-p\left(y=1|x\right)}\text{i}\text{.e}\text{., }p\left(y=1\text{|}x\right)=\;\frac{1}{1+e^{-\left(w^{T}x+b\right)}}$$ (1)

Random Forest is an integrated algorithm composed of decision trees, which achieves excellent performance in many applications. Decision tree is a supervised learning algorithm based on “if-then-else” rules. When we perform the classification task, the input samples are classified by each decision tree separately. And each decision tree will get its own classification result. Those decision trees form the random forest, and it will ensemble all prediction results, and output the label with the most consistent evidence.

Support vector machines (SVM) is a binary classification model. Its basic model is a linear classifier featured with the largest interval between two classes in the feature space. Kernel techniques can be applied to SVM, which makes it a non-linear classifier. The learning strategy of SVM is to maximize the interval, which can be formalized as a problem of solving convex quadratic programming. As shown in Eq. 2, the SVM model is to construct the hyperplane ( ω is the variable coefficient, γ is the constant), so that the labels of the samples can be divided correctly.

$$\;\omega x^{T}+\;\gamma =0$$ (2)

### Metrics for Evaluating Prediction Performance

In the task of binary classification, samples in the test set can be separated into four classes: true positive (TP), true negative (TN), false positive (FP), and false negative (FN). And the sample size of the test set (*N*) equals to the sum of TP, TN, FP, and FN. Based on these measures, we used four metrics to evaluate the prediction performance: accuracy (ACC), area under the receiver operating characteristic curve (AUROC), area under the precision and recall curve (AUPRC) and F1 score. The accuracy is defined as the ratio of number of correctly predicted samples (TP + TN) and the sample size of the test set (*N*). However, ACC is not robust in study with unbalanced samples, which means there is only a small number of positive/negative sample. The other three metrics can solve this problem to some extent. The PR curve is defined based on precision and recall which are defined in Eqs 3, 4, respectively. The precision and recall are on *y* and *x*-axis respectively. Since there are *N* possible thresholds of prediction probability, there would be *N* points, i.e., (precision, recall) on the PR curve.

$$precision=\;\frac{TP}{TP+FP}$$ (3)

$$recall=\;\frac{TP}{TP+FN}$$ (4)

Similarly, the ROC is defined based on true positive rate (TPR) and false positive rate (FPR), which are defined in Eqs 5, 6 respectively. In ROC, the TPR and FPR are on *y* and *x*-axis respectively. F1 score is a combination of precision and recall, which is defined in Eq. 7.

$$TPR=\;\frac{TP}{TP+FN}$$ (5)

$$FPR=\;\frac{FP}{TN+FP}$$ (6)

$$F1=\;\frac{2*precision*recall}{precision+recall}$$ (7)

The area under ROC and PRC (AUROC and AUPRC) are widely used to compare the performance of different classifiers. Given a series of points $\left\{\left(x_{1},y_{1}\right),\left(x_{2},y_{2}\right),\ldots \ldots ,\left(x_{n},y_{n}\right)\right\}$ on the ROC or PRC curve, the area under the curve (AUC) can be approximately computed by Eq. 8.

$$AUC=\frac{1}{2}\overset{n-1}{\underset{i=1}{\sum }}\left(x_{i+1}-x_{i}\right)\cdot \left(y_{i}+y_{i+1}\right)$$ (8)

## Results and Discussion

### Datasets

We first downloaded the diabetes mellitus associated genes from DisGeNet database (as of June 2021, UMLS CUI: C0011849). 2,803 genes were recorded in this database, and each gene was assigned with a gene-disease association (GDA) score, indicating the levels of evidence. The GDA score takes into account the number and type of sources (level of curation, organisms), and the number of publications supporting the association. After filtering GDA score with threshold set to 0.1, there were 476 genes left that were used for model training in the downstream prediction.

The protein-protein interaction network was obtained from Menche et al.’s work (Menche et al., 2015). This PPI network consists of multiple sources of protein interactions, such as regulatory interactions, yeast two-hybrid high-throughput interactions, literature curated databases, metabolic enzyme-coupled interactions, protein-protein complexes, etc. By combining those interactions, we obtained this PPI network of 13,460 proteins and 141,296 interactions.

### Network Representation Learning Using DeepWalk, LINE, and Node2vec

We extracted the node features of the PPI network using the technique of network representation learning or graph embedding, which maps the topological features of nodes in the network into the embedding space. To choose a proper method, three cutting-edge network representation learning methods were used for feature extraction. And we compared their performance using five-fold cross-validation. To balance the sample size of positive samples and negative samples, we randomly selected the same number of nodes not labeled as disease genes as negative samples.

We run these methods on the PPI network and generate features with 512 dimensions. Then the features were further processed by a stacked autoencoder with three levels, which will reduce noises and generate latent features. The 512-dimensional features were converted to 64-dimensional features using this autoencoder. And SVM was used for final classification using the same setting parameters.

**FIGURE 2:**
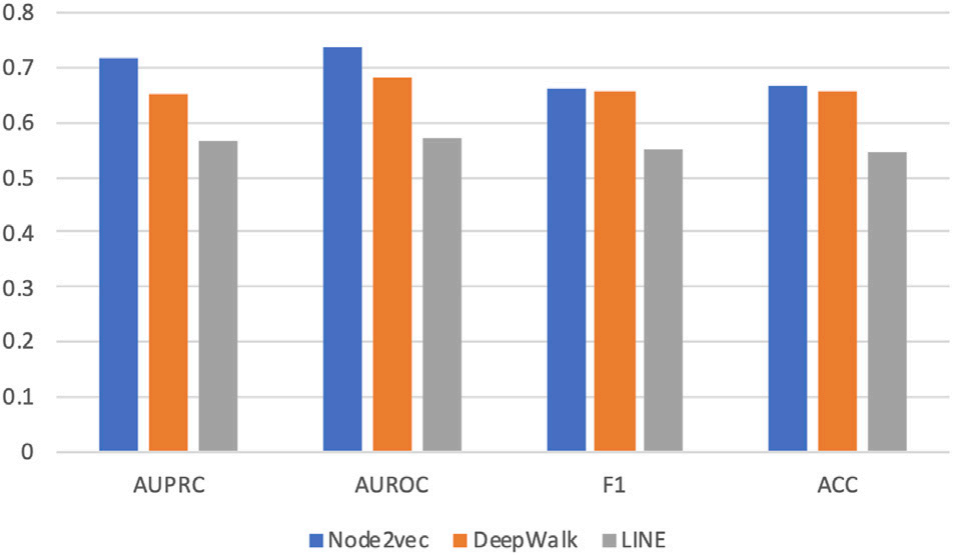
Prediction performance in five-fold cross validation based on three graph embedding methods. Three different graph embedding methods are compared: DeepWalk, LINE, and Node2vec. Four metrics are used for performance evaluation: AUROC, AUPRC, F1 score, and accuracy (ACC).

Figure 2 shows the average AUROC, AUPRC, F1 score, and accuracy (ACC) values of three methods achieved in this experiment. We can see that Node2vec achieves the best performance under all metrics. And DeepWalk is the second-best method. This is easy to understand because Node2vec improves DeepWalk by a biased random-walk strategy (see details in Methods).

### Feature Dimension Affects Prediction Performance

As a non-end-to-end model, our framework first generates features of network nodes and then predicts disease-associated genes based on SVM. All of the three network-representation-learning methods mentioned above are based on a skip-gram neuron network model, where the dimension of output features equals the number of neurons in the hidden layer of skip-gram neuron network. To explore the impact of feature dimensions on our predicting framework, we compared the performance of the representation learning methods with various dimensional features extracted from the PPI network. Those features were all converted to 64-dimensional features using the stacked autoencoder described above, followed by the SVM classifier under the same settings (RBF kernel and other settings in default).

**FIGURE 3:**
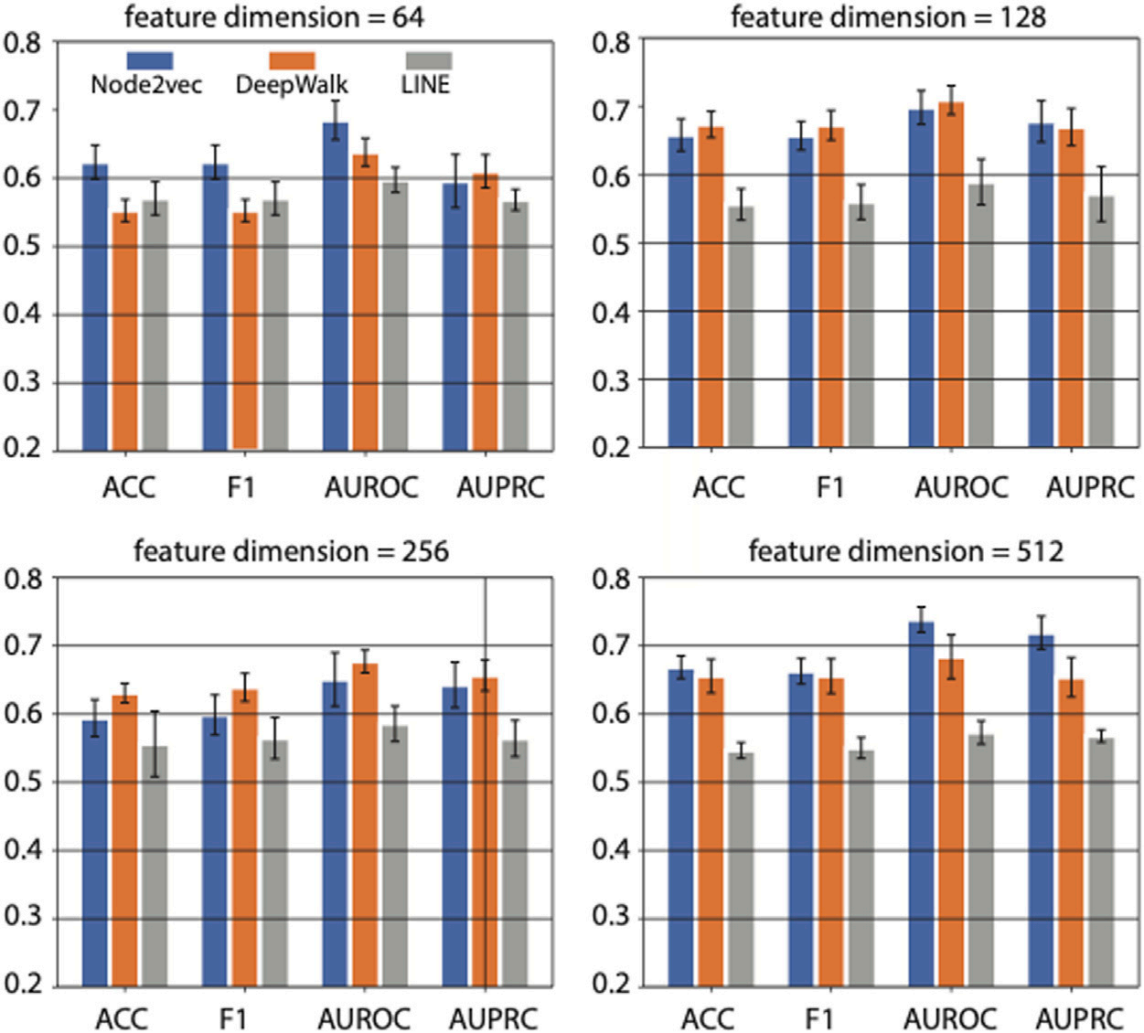
The effects of feature dimension on prediction performance. Four feature dimensions (i.e., 64, 128, 256, and 512) generated by graph embedding methods are used for comparison. Three different graph embedding methods are also compared.

Based on five-fold cross-validation, we got the results shown in Figure 3. The four sub-panels in Figure 3 represent the prediction performance on diabetes genes using different feature dimensions (i.e., 64, 128, 256, and 512 feature dimensions) generated by three network representation learning methods. The average AUROC, AUPRC, F1 score, and ACC values were compared.

When the feature dimension equals 64, Node2vec achieved the best performance in ACC, F1 score, and AUROC. And LINE achieved the best performance in AUPRC and the second-best performance on ACC and F1 score. While as the feature dimension increased to 128 and 256, the DeepWalk achieved the best performance, and Node2vec achieved the second-best rank. However, The Node2vec achieved the maximum AUROC (0.74) and AUPRC (0.72) scores with 512 feature dimensions compared with other methods in various feature dimensions. In summary, the feature dimension and network representation learning method both affect the prediction performance in a task-dependent way. In our case, i.e., predicting genes associated with diabetes mellitus, we choose Node2vec as the method of feature learning from PPI network, and output 512-dimensional features in downstream analysis.

### Exploring the Effect of Hyper-Parameters in Node2vec and Different Classifiers

**FIGURE 4:**
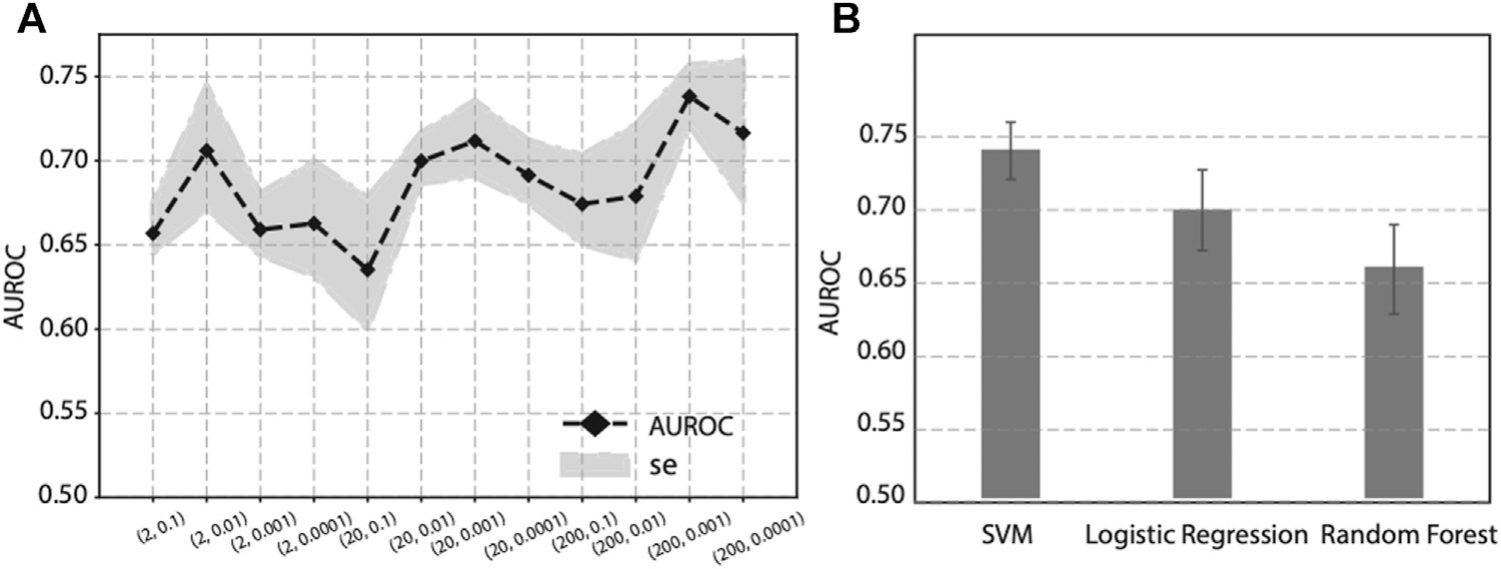
Effect on prediction performance by hyper-parameters in Node2vec and different machine learning classifiers. **(A)** Prediction performance under various *p* and *q* values in Node2vec. **(B)** Prediction performance of SVM, Logistic regression and Random Forest in five-fold cross validation.

As previous publications have pointed out, the hyper-parameter *p* and *q*, in Node2vec have potential influence to feature learning and downstream analysis. To optimize the two parameters, we performed a grid search on *p* and *q*, and calculated the corresponding performance. Since p controls the random walk to visit new nodes or visited nodes, we set *p* in a larger manner to encourage the random walk to visit new nodes, and we choose *p* ∈ (2, 20, 200). And q controls the random walk towards a BFS or DFS graph search. To let the random walk be biased to a DFS search, we set *q* ∈ (0.1,0.01, 0.001, 0.0001). The performance of various *p* and q values is shown in Figure 4A. It seems there is not a linear relationship between (*p*, *q*) values and the performance. As we can see, when *p* = 200 and *q* = 0.001, it achieves the best performance (AUROC = 0.74) on this specific task, i.e., prediction genes associated with diabetes mellitus. Since the best combination of (*p*, *q*) values varies from study to study, it is recommended to perform a grid search to find the best hyperparameters.

To evaluate the effect of different classifiers, we compared SVM with two other widely-used classifiers: Logistic regression and Random Forest. Using the same features obtained from Node2vec followed by a stacked autoencoder, we compared the prediction performance of SVM, Logistic regression, and Random Forest in five-fold cross-validation. The results are shown in Figure 4B, where we can see SVM achieves the best performance than Logistic regression and Random Forest. Based on this analysis, our prediction model will use SVM as classifier to predict genes associated with diabetes mellitus.

### Top Genes Predicted to Be Associated With Diabetes Mellitus

To discover novel genes associated with diabetes mellitus, we predicted all unlabeled genes in the PPI network using the final trained model. The model uses Node2vec (with *p* = 200 and *q* = 0.001) to extract node features in 512-dimension followed by a three-layer autoencoder to compress the feature to 64-dimension, and SVM is applied to predict the possibility of unlabeled genes to be a diabetes gene. The SVM model was trained using all the 476 genes labels as disease-related. Then all the unlabeled genes were predicted by SVM. We ranked the gene predicted by our methods and listed the top 15 genes in Table 1. The size of the top 15 genes is artificially set.

**TABLE 1:** Top 15 genes predicted associated with diabetes mellitus.

Gene id	Gene name	Gene description	Score
331	BIRC4	X-linked inhibitor of apoptosis	0.78
7098	TLR3	Toll like receptor 3	0.77
55905	ZNF313	Ring finger protein 114	0.76
8915	BCL10	BCL10 immune signaling adaptor	0.76
3654	IRAK1	Interleukin 1 receptor associated kinase 1	0.75
3659	IRF1	Interferon regulatory factor 1	0.75
84270	CARD19	Caspase recruitment domain family member 19	0.75
64320	RNF25	Ring finger protein 25	0.75
340061	TMEM173	Stimulator of interferon response CGAMP interactor 1	0.74
59307	SIGIRR	Single ig and TIR domain containing	0.74
9451	EIF2AK3	Eukaryotic translation initiation factor 2 alpha kinase 3	0.74
5608	MAP2K6	Mitogen-activated protein kinase 6	0.73
51135	IRAK4	Interleukin 1 receptor associated kinase 4	0.73
220885	RPSAP15	Ribosomal protein SA pseudogene 15	0.73
9344	TAOK2	TAO kinase 2	0.73

Researchers have delineated the relevance of some predicted genes to diabetes mellitus. Zhou et al. (2017), evaluated the gene-environment interactions and haplotype associations and extrapolated the pathogenic role of genetic variants in the TLR3-TRIF-TRAF3-INF-β in causing type 2 diabetes mellitus. Al Dubayee et al. (2021), examined the increased expression of BCL10 and reduced expression of caspase-7 from peripheral blood mononuclear cells of diabetic individuals during the apoptosis in insulin resistance, which reveals close relationship between BCL10 gene and diabetes mellitus. Maikel et al. (Colli et al., 2018), utilized immunofluorescence to discern the positive correlation between expression of PDL1 and IRF1, based on the fact that PDL1 expression is elevated in insulin-containing islets of individuals with type 1 diabetes, IRF1 and Diabetes Mellitus show a high probability of interaction.

**FIGURE 5:**
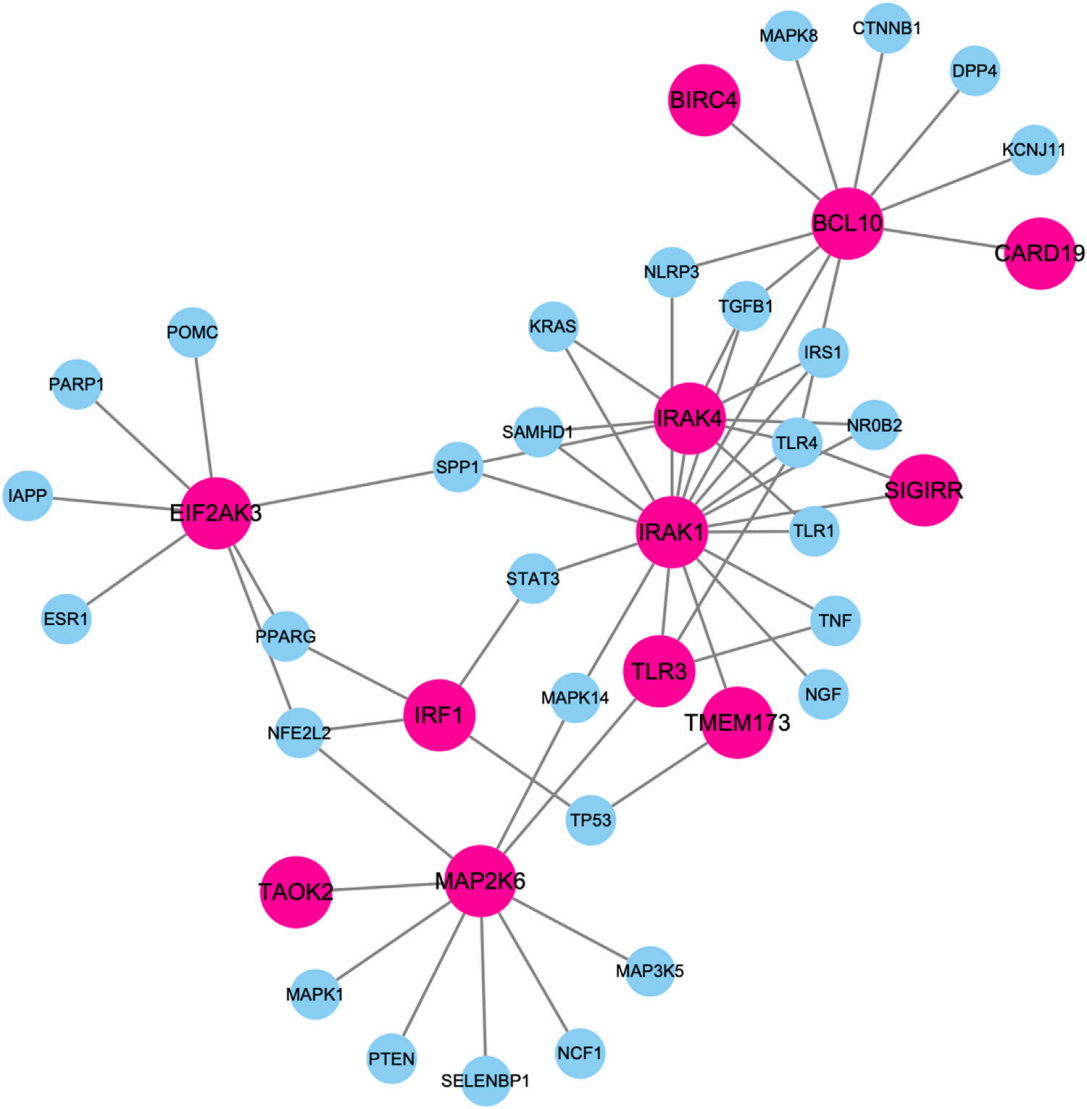
Largest component of PPI subnetwork among these top-predicted genes and known genes associated with diabetes mellitus. Nodes in pink represent top predicted genes. Nodes in blue represent know diabetes genes.

Figure 5 shows the largest component of PPI subnetwork among these top-predicted genes and known genes associated with diabetes mellitus. Those predicted genes are closely connected with known diabetes genes in the database. For example, IRAK1 and IRAK4 have the highest degrees connecting both known genes and predicted genes. It has been shown that deletion of IRAK1 improves glucose tolerance by elevating insulin sensitivity (Sun et al., 2017). IRAK4 inhibitors can block MyD88 dependent signaling, which contributes to the pathogenesis of type I diabetes (Sabnis, 2021).

### Functional Enrichment Analysis of the Predicted Genes

**FIGURE 6:**
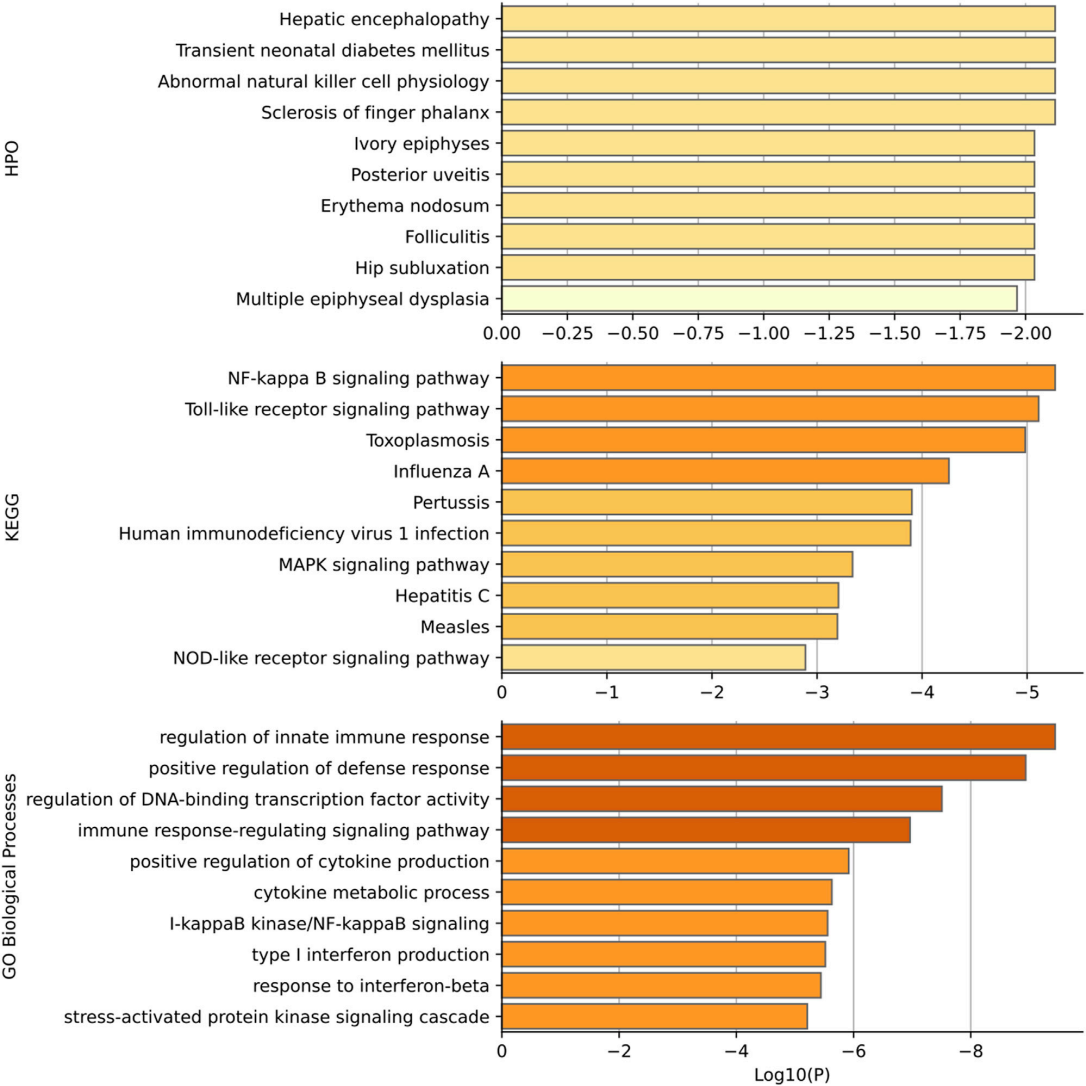
Functional enrichment results based on HPO, KEGG, and GO. *p*-values are shown in log scale and only top 10 terms are shown in each category.

Gene set enrichment analysis has been performed for the top 15 genes predicted to be related to diabetes mellitus. Gene functional categories in Human Phenotype Ontology (HPO), KEGG, and GO biological process were used for over-representation analysis using WebGestaltR (Liao et al., 20192019). The top enrichment terms are shown in Figure 6. Our predicted genes have shown over-representation in genes of the HPO term “transient neonatal diabetes mellitus” with suggestive *p*-value < 0.01. The top HPO term enriched was “hepatic encephalopathy,” and it has been shown that diabetes mellitus plays a role in hepatic encephalopathy by releasing and enhancing the inflammatory cytokines (Ampuero et al., 2013). In KEGG enrichment results, the term “NF-kappa B signaling pathway” achieves the best significance with *p*-value < 5*10^–5^. Romeo et al. (2002) has shown that diabetes and high glucose can induce the activation of nuclear factor-kB (NF-kappa B), which regulates a proapoptotic program in retinal pericytes. The second term is “Toll-like receptor signaling pathway diabetes” with enrichment *p*-value < 5*10^–5^. Dasu and Martin (2014) has shown the increased toll-like receptors (TLRs) expression and activation contribute to the hyper inflammation in human diabetic wounds. The third enriched term is “toxoplasmosis”. There have been findings that patients with toxoplasmosis are more susceptible to be diabetics than those without toxoplasmosis, suggesting a role of toxoplasmosis in diabetes mellitus (Shirbazou et al., 2013). Most enriched terms in GO are related with the immune response. And it has been well established that patients with diabetes mellitus have more susceptibility to infections (Berbudi et al., 2020). The high blood glucose levels, as well as the inflammatory mediators produced by adipocytes and macrophages, can result in the immune response (Geerlings and Hoepelman, 1999).

## Conclusion

Diabetes mellitus has widely affected the population in the world, without knowing the underlying mechanism. Discovering genes associated with diabetes will pave the way for developing novel efficient therapies. In this work, we designed a computational framework for diabetes gene prediction based on graph embedding techniques. This framework consists of three main steps: network feature extraction based on graph embedding methods; feature denoising and regeneration using stacked autoencoder; and disease-gene prediction based on machine learning classifiers. By comparing with different graph embedding methods and widely-used machine learning classifiers, we proved the efficiency and accuracy of our method. By applying this method to diabetes gene discovery, we found novel genes that have been reported in publications with clear association evidence but not recorded in the database. Through functional enrichment analysis based on Human Phenotype Ontology (HPO), KEGG, and GO biological process, we found the top predicted genes are enriched in multiple terms that have been proved to have a role in diabetes mellitus. Our computational method may also benefit gene discoveries for other complex diseases.

## Data Availability

The original contributions presented in the study are included in the article/supplementary material, further inquiries can be directed to the corresponding authors.
